# 2497. Analysis of Social Determinants of Health (SDOH) and their Implications for Hepatitis C Treatment in People Who Inject Drugs: The Case of Baltimore

**DOI:** 10.1093/ofid/ofad500.2115

**Published:** 2023-11-27

**Authors:** Luis A Gonzalez Corro, Oluwaseun Falade-Nwulia, Kathleen R Page, Gregory Lucas

**Affiliations:** Johns Hopkins, Baltimore, Maryland; Johns Hopkins University, Baltimore, MD; Johns Hopkins School of Medicine, Baltimore, MD; Division of Infectious Diseases, Johns Hopkins University School of Medicine, Baltimore, Maryland

## Abstract

**Background:**

Of 3.5 million people living with HCV in the US, nearly 60% are people who inject drugs (PWID). Despite the availability of a cure for HCV, uptake remains low in PWID. We set out to study the social determinants of health (SDOH) that impact the HCV care cascade.

**Methods:**

We conducted a secondary analysis of baseline data from 720 PWID in a cluster-randomized trial. We recruited PWID via an outreach van from 12 drug-affected areas in Baltimore City. Inclusion criteria included injection in the prior month or needle sharing in the past 6 months. An intake visit included a survey and a blood draw for HCV testing (serology reflexed to HCV RNA). Focusing on SDOH, we analyzed factors related to self-report of i) awareness of HCV infection (in the subgroup with active or treated HCV) and ii) prior HCV treatment (in the subgroup that was aware of their HCV infection). We used descriptive statistics and logistic regression for statistical analyses.

**Results:**

The 342 PWID included in our study had a median age of 52 years and most were male and Black. 53% injected daily and 67% reported receptive needle sharing in the past 6 months. Of 251 who were aware of their HCV infection, 34% reported treatment.

Women were more likely to be aware of their status but less likely to be treated. Older age and Black race were both associated with increased odds of HCV treatment (with evidence of confounding as the median age was 8 years lower in white vs. Black people). Having a primary care provider and HIV+ status (a likely proxy for care access) were both significantly tied to higher odds of awareness and of treatment. Unhoused people had 51% lower odds of HCV treatment (vs. stably housed). Finally, we found a positive link to social support: people who reported that other PWID had shared their HCV+ status with them had 2.3-fold higher odds of awareness of their own status. Similarly, people with ≥ 3 (median) close people to talk to had over twice the odds of HCV treatment (vs. < 3) in an unadjusted model.

**Figure:**
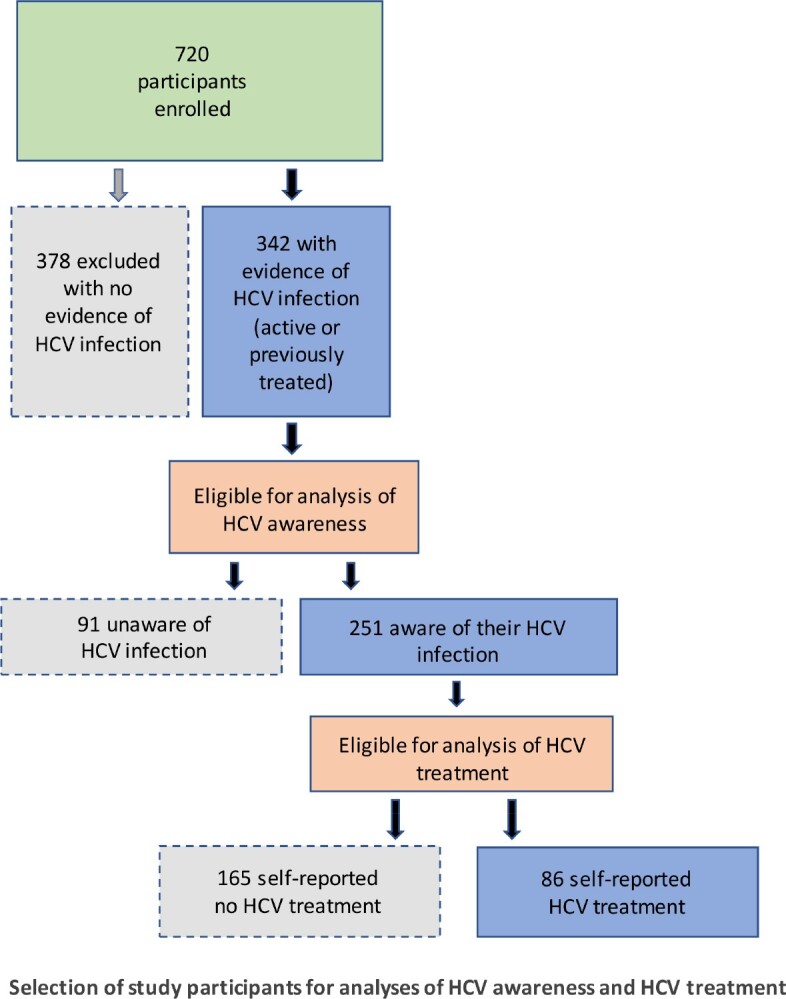

**Figure:**
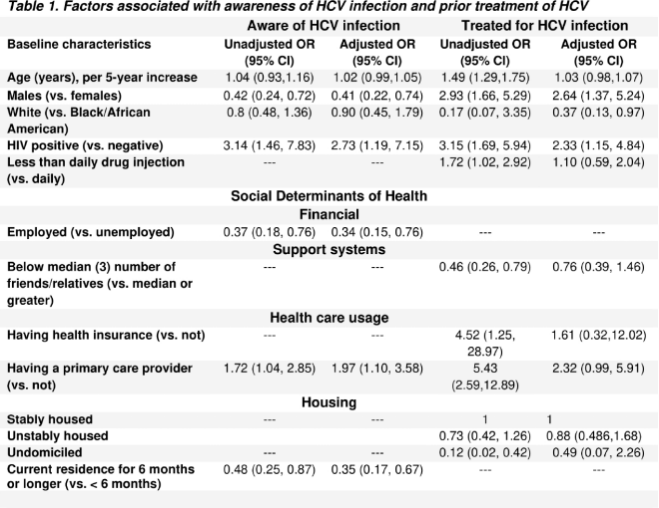

**Conclusion:**

In this study of street recruited PWID, we observed a lower treatment uptake in women despite greater HCV infection awareness. SDOH categories with significant associations to the HCV care cascade included finances, stigma, support system, healthcare usage, and housing status. Strategies to not only identify but address SDOH are vital to end HCV.

**Disclosures:**

**Oluwaseun Falade-Nwulia, MBBS ,MPH**, Abbvie Inc: Grant/Research Support|Gilead Sciences: Advisor/Consultant

